# Facteurs prédictifs de l’encéphalopathie hépatique au cours de l’atteinte hépatique aiguë sévère

**DOI:** 10.11604/pamj.2022.42.323.30089

**Published:** 2022-08-31

**Authors:** Amal Khsiba, Samir Bradai, Moufida Mahmoudi, Asma Ben Mohamed, Mouna Medhioub, Lamine Hamzaoui, Mohamed Mousadek Azouz

**Affiliations:** 1 Gastroenterology Department, Mohamed Tahar Maamouri Hospital, Nabeul, Tunisia

**Keywords:** Atteinte hépatique aiguë sévère, insuffisance hépatique aiguë, encéphalopathie hépatique, pronostic, Severe acute liver injury, acute liver failure, hepatic encephalopathy, prognosis

## Abstract

**Introduction:**

l´atteinte hépatique aiguë sévère (AHAS) est une inflammation aiguë du foie avec des perturbations des marqueurs d´atteinte hépatique et des signes d´insuffisance hépatocellulaire (ictère et INR supérieur à 1,5) selon définition de l´association européenne pour l'étude du foie.Le facteur qui conditionne le pronostic de l´AHAS reste l´apparition d´une encéphalopathie hépatique (EH). L´objectif de ce travail est de rechercher les facteurs prédictifs du développement de l´EH au cours de l´AHAS.

**Méthodes:**

il s´agit d´une étude observationnelle rétrospective entre janvier 2000 et décembre 2019. Nous avons réalisé une étude analytique comparant les deux groupes en fonction de l´apparition ou non d´une EH.

**Résultats:**

cinquante-neuf patients ont été colligés. La survenue d'une EH était observée chez 15 patients (25,4%). Les facteurs prédictifs de la survenue d´une EH en analyse univariée étaient un délai de consultation supérieur à 9 jours, un taux d´INR supérieur à 2,45, un taux de bilirubine supérieur à 230 μmol/l, créatininémie supérieur à 60,5 μmol/l, un taux d'urée supérieur à 5,5 mmol/l et un score MELD supérieur à 26,5 (p=0,023, p = 0,017, p = 0,0001, p=0,049, p = 0,0001, p = 0,0001 respectivement). L´hépatite auto-immune et la cause indéterminée étaient associées à l´apparition d´une EH (respectivement p=0,003 et p=0,044). En analyse multivariée, l´étiologie auto-immune et un taux d´urée supérieur à 5,5 mmol/l étaient significativement associés à la survenue d´une EH.

**Conclusion:**

la survenue de l´EH est le résultat de l´interférence de plusieurs facteurs associant des paramètres biologiques comme l´INR, la bilirubinémie, la fonction rénale et l´étiologie en cause.

## Abstract (en)

**Introduction:**

severe acute liver injury (SALI) formerly known as severe acute hepatitis is an acute inflammation of the liver with markers of liver injury (elevated transaminases) and signs of hepatocellular failure (jaundice and INR greater than 1.5) according to the latest definition of the European Association for the Study of the Liver (EASL). An important prognostic factor in SALI is the development of hepatic encephalopathy (HE) and thus its progression to acute liver failure (ALF), formerly known as acute severe hepatitis. The purpose of this study is to investigate factors predicting the development of hepatic encephalopathy during SALI.

**Methods:**

we conducted a retrospective study of patients treated for SALI between January 2000 and December 2019. We divided patients into two groups depending on whether hepatic encephalopathy occurred. We performed an analytical study comparing the two groups according to their epidemiological, biological and evolutionary data.

**Results:**

data from the medical records of fifty-nine patients were collected. A virus was the most frequent cause (63%). Hepatic encephalopathy occurred in 15 patients (25.4%). Factors predicting the development of HE in univariate analysis were a delay in consultation of more than 9 days, an INR level of more than 2.45, a bilirubin level of more than 230 μmol/l, creatinine greater than 60.5 μmol/l, urea greater than 5.5 mmol/l and MELD score greater than 26.5 (p=0.023, p=0.017, p=0.0001, p=0.049, p=0.0001, p=0.0001 respectively). Autoimmune hepatitis and an undetermined cause were associated with the development of HE (p=0,003 and p=0,044, respectively). In multivariate analysis, autoimmune aetiology and a urea level above 5.5 mmol/l were significantly associated with the occurrence of HE. No statistically significant differences were found between the two groups with regard to age, sex and diabetes.

**Conclusion:**

SALI is a rare disease, mainly due to a virus in our country. Predictive factors of HE are important for early identification of patients at risk of adverse outcomes.

## Introduction

L´atteinte hépatique aiguë sévère (AHAS) anciennement appelée hépatite aiguë sévère est une inflammation aiguë du foie en l´absence d´une pathologie hépatique préexistante. La définition selon la dernière définition de l´association européenne pour l'étude du foie (EASL) est biologique associant des marqueurs d´atteinte hépatique (élévation des transaminases) et des signes d´insuffisance hépatocellulaire (ictère et International normalized ratio (INR) supérieur à 1,5) [1]. L´apparition d´une encéphalopathie hépatique (EH) traduit le passage vers une insuffisance hépatique aiguë (IHA) anciennement appelée hépatite aiguë grave. Ce passage représente un tournant évolutif dans la maladie [1]. En effet, l´IHA est un état de défaillance hépatique aiguë compliquée d´une EH, associé à coagulopathie sévère et une détérioration systémique résultant d´un syndrome de réponse inflammatoire systémique [1]. Tous les patients atteints d'une AHAS rétablissent le plus souvent spontanément ou après un traitement spécifique, contrairement à l´IHA qui présente un taux de mortalité élevé atteignant 30% en l´absence d´une transplantation hépatique [2]. Le diagnostic de l´IHA à un stade précoce avant l´apparition de signes neurologiques est une étape importante dans la prise en charge des AHAS pour sélectionner les malades à risque d´une évolution défavorable et les orienter précocement à un centre spécialisé à proximité d´une unité de transplantation hépatique. Mais, les facteurs prédictifs de cette transition de l´AHAS à l´IHA restent encore peu connus et étudiés. L´association européenne pour l'étude du foie (EASL) souligne l´importance de prédire ces facteurs dans des futures études pour mieux comprendre l´histoire naturelle de l´AHAS [1]. L´objectif de notre travail était d´identifier les facteurs prédictifs d´une encéphalopathie hépatique au cours de l´AHAS.

## Méthodes

**Cadre et type d´étude:** il s´agit d´une étude rétrospective observationnelle de type descriptive et analytique réalisée au service d´Hépato-gastro-entérologie de l´hôpital Mohamed Taher Maamouri de Nabeul entre le 1^er^ janvier 2002 et le 31 décembre 2019 afin d´identifier les facteurs prédictifs d´une encéphalopathie hépatique au cours de l´AHAS.

**Population de l´étude:** On a inclus les patients hospitalisés pour la prise en charge d´une AHAS définie par l´élévation des marqueurs d´atteinte hépatique (élévation des transaminases) et des signes d´insuffisance hépatocellulaire (ictère et International normalized ratio (INR) supérieur à 1.5) selon l´EASL [1]. Les critères d´exclusion étaient l´atteinte hépatique aiguë non sévère (INR < 1,5) et les patients avec une poussée aiguë sur une hépatopathie chronique connue. Nous avons divisé les patients en deux groupes selon l´apparition d´une encéphalopathie hépatique ou non Groupe 1 (G1): ceux n´ayant pas présenté une EH et groupe 2 (G2): ceux ayant évolué vers une EH.

**Recueil des données:** on a relevé pour toute la population d´étude:

**Les caractéristiques épidémio-cliniques**: âge, sexe, les antécédents notamment le diabète, les habitudes essentiellement l´alcoolisme chronique, le type de la symptomatologie, le délai de la consultation par rapport à l´apparition des symptômes et les données de l´examen clinique en particulier la mesure de la flèche hépatique, la recherche d´une ascite ou d´une splénomégalie et l´examen neurologique à la recherche de signes d´EH. Nous avons précisé le délai de l´apparition de l´EH par rapport à l´ictère et son grade selon la classification de West Haven [3].

Les étiologies en cause après des explorations à visée étiologique comportant en premier lieu la recherche d´une cause virale par la pratique des sérologies virales A (IgM anti VHA), B (Antigène HBs et IgM anti HBc), C (anti VHC et la PCR du VHC) et E (IgM anti-VHE et ARN viral). En cas de négativité d´une cause virale, une autre étiologie sera recherchée par un interrogatoire exhaustif à la recherche de prise de médicament ou de substance hépatotoxique, un bilan immunologique comportant les anticorps anti-nucléaires (ANN), les anticorps antimuscle lisse (AML) et les anticorps anti LKM avec le dosage pondéral des immunoglobulines, un bilan cuprique (céruléoplasmine, cuprémie, cuprurie) en cas de suspicion d´une maladie de Wilson, la recherche des virus non hépatotrope tels que le virus d'Epstein-Barr (EBV), cytomégalovirus (CMV), virus de l'herpès simplex (HSV) et les infections par le virus varicelle-zona (VZV), une échographie abdominale avec Doppler des veines sus hépatiques (VSH) en cas de suspicion de syndrome de Budd-Chiari (SBC) complétée par un angioscanner abdominal afin de confirmer le diagnostic, une échographie transthoracique (ETT) en cas de suspicion d´une étiologie ischémique due à une défaillance cardiaque. La ponction biopsie hépatique (PBF) est réalisée en l´absence de contre-indication si une hépatite auto-immune est suspectée ou si le bilan étiologique est négatif. Une origine non déterminée est considérée si le bilan étiologique réalisé est négatif.

**Les paramètres biologiques suivants:**

**Le bilan hépatique**: aspartate amino transférase (ASAT), alanine amino transférase (ALAT), le taux de la bilirubine, des phosphatases alcalines (PAL).

**Le bilan rénal:** taux d´urée, et de la créatinine. La numération et formule sanguine (globules blancs, taux de plaquettes). Le bilan de l´hémostase: le taux de prothrombine (TP), l´international normalized ratio (INR).

Nous avons calculé pour tous les patients le score « Model for End-stage Liver Disease » (MELD) selon la formule: MELD Score = (0,957 * Créatinine sérique) + (0,378 * Bilirubine sérique) + (1,120 * international normalized ratio) + 0, 643 [4,5].

**Source de données:** le recueil a été fait à partir des dossiers médicaux complets et exploitables.

**Collecte et analyse des données:** la collecte et l'analyse des données ont été faites à partir d'une fiche d'enquête.

**Biais:** pour réduire le risque des biais d'information et d'enregistrement, les données ont été recueillies par deux personnes et les données finales ont résulté de la fusion des listes finales.

**Taille de la population d´étude:** la taille de l'échantillon a été déterminée par le nombre des patients ayant satisfait aux critères d'inclusion sans avoir d'élément des critères d'exclusion.

**Analyse statistique:** la saisie des données a été réalisée en utilisant le logiciel SPSS 22. Les variables qualitatives ont été exprimées en pourcentage et les variables quantitatives en moyenne avec écart type. La recherche des variables associées à la survenue d´une EH a été d´abord faite par une étude univariée, au moyen du test de Student pour les variables quantitatives, du test de chi-deux de Pearson et du test exact de Fisher pour les variables qualitatives, après avoir transformé les variables quantitatives en variables qualitatives à deux modalités selon les valeurs seuils identifiées par les courbes *Receiver Operating Characteristics (ROC)*. Après avoir vérifié que l´aire sous la courbe (ASC) est significativement supérieure à 0,5, nous avons choisi, comme seuil, la valeur de la variable qui correspondait au meilleur couple « sensibilité spécificité ». L´analyse univariée a été complétée par une analyse multivariée en régression logistique afin d´identifier les facteurs liés de façon indépendante à la survenue d´une EH. Une valeur p inférieure à 0,05 était considérée comme significative.

**Considérations éthiques:** l´analyse des données s´est déroulée dans le respect des valeurs de l´éthique médicale, de l´anonymat et de la confidentialité.

## Résultats

**Caractéristiques épidémiologique et clinico-biologique:** nous avons inclus 59 patients âgés en moyenne de 36±18 ans. Le sex ratio était de 0,73 avec 25 hommes et 34 femmes. Six patients étaient diabétiques (10%). La notion d´alcoolisme chronique était présente chez 10 patients (17%). Le délai moyen de consultation était de 9 jours ± 8 jours. L´ictère cutanéo-muqueux est le symptôme le plus fréquemment retrouvé chez 54 patients (92%), suivi par les douleurs abdominales (44%) et la fièvre (42%). Un seul malade a présenté un saignement extériorisé sous forme d´épistaxis (1,6%). Une éruption cutanée a été trouvée chez un seul patient (1,6%). L´examen clinique a montré une hépatomégalie chez 8 patients (13%), un seul cas d´atrophie hépatique (2%), une splénomégalie chez 3 patients (5%) et une ascite chez 5 cas (8%). Les caractéristiques épidémiologiques et clinico-biologiques des patients sont détaillées dans le Tableau 1. Quinze malades avaient présenté une EH (25,4%). Le délai moyen d´apparition de l´EH par rapport l´ictère était de 11 jours (2-30 jours). Le grade de l´EH selon la classification de West Haven était: Grade I dans 2 cas, grade II dans 6 cas et grade III dans 7 cas.

**Tableau 1:** caractéristiques de la population étudiée

Paramètres	Patients (n=59)
Age (ans)	36 +/- 18
Sex ratio	0,73 (Homme/Femme: 25/34)
Alcoolisme chronique	10 (17%)
Diabète	6 (10%)
Délai moyen de consultation (jours)	9 +/- 8
Ictère	54 (92%)
Splénomégalie	3 (5%)
Ascite	5 (8 %)
Flèche hépatique (cm)	11,75 +/-1,3
ASAT (UI/L)	1361 +/- 1092
ALAT (UI/L)	1470 +/-1246
PAL (UI/l)	168,9 +/- 202,75
Taux de la bilirubine (μmol/l)	201,75 +/- 145 ,45
TP (%)	38 +/- 10,6
INR	2,8 +/- 1,5
Taux des leucocytes (/mm3)	8817 +/- 6234,5
Taux des plaquettes (/mm3)	435214,5 +/- 104397
Taux de la créatinine (μmol/l)	71 +/- 48,8
Taux de l´urée (mmol/l)	6,68 +/ 6,4

ASAT: Aspartate aminotransférase, ALAT: Alanine aminotransférase, PAL: Phosphatases alcalines, TP: Taux de prothrombine, INR: International normalized ratio

**Caractéristique étiologique:** concernant l´étiologie de l´atteinte hépatique, l´origine virale était la cause la plus fréquente (64,2%): virus de l´hépatite A (VHA) chez 15 patients (25,4%), virus de l´hépatite B (VHB) chez 18 patients (30,5%), hépatite virale C (VHC) chez 4 patients (6,7%) et une infection par herpès simplex virus (HSV) dans 1 cas (1,6 %). Une lésion hépatique d´origine médicamenteuse a été observée dans 8 cas (13,55%), une hépatite auto-immune (HAI) dans 4 cas (6,7%), un syndrome de Budd-Chiari aigu (SBC) dans 2 cas (3,2%), une insuffisance cardiaque aiguë 2 cas (3,2%) et les métastases hépatiques dans 1 cas (1,6%). L´étiologie était de cause indéterminée chez 4 patients (6,7%).

### Les facteurs prédictifs d´apparition d´une EH

**Figure 1:**
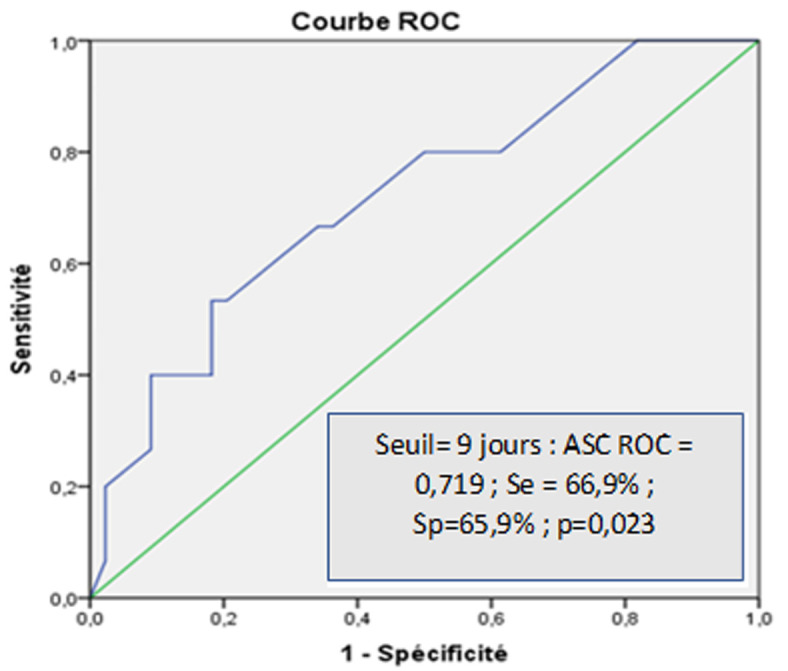
courbe ROC du délai de la consultation pour l´évaluation du risque de l´encéphalopathie hépatique

**Facteurs épidémio-cliniques:** l'âge des patients du groupe 2 était plus avancé (G1 : 33 Vs G2: 41) mais la différence n´était pas statistiquement significative (p=0,143). On n´a pas trouvé une différence significative entre les deux groupes concernant le sexe (p=0,315). L´alcoolisme était présent chez 23% des sujets du G1. Par contre, il était absent dans le G2. Une différence statistiquement significative a été constatée entre les deux groupes (23% vs 0%; p = 0,039). Le diabète était plus fréquemment observé chez les sujets du G1 avec une différence non significative (14% vs 0%; p = 0,157). La comparaison de délai de consultation entre les deux groupes a montré un délai plus long chez les patients du G2 (G1: 7 vs G2: 14 jours) avec une différence significative (p=0,004). La représentation graphique de la corrélation entre le délai de la consultation et le risque de développement d´une EH a suggéré un risque plus important à partir d´un seuil de 9 jours (aire sous la courbe ROC = 0,719 [IC95%, 0,569-0,869] avec une sensibilité à 66,9% et une spécificité à 65,9%, p=0,023) (Figure 1). Les données de l´examen clinique, notamment la flèche hépatique, la présence de splénomégalie ou d´ascite, étaient comparables entre les deux groupes.

**Facteurs biologiques:** biologiquement, les patients ayant évolué à l´EH avaient un INR, un taux de bilirubine, d´urée et de créatinine significativement plus élevés (respectivement p= 0,015, p=0,0001, p =0,007, p=0,003) avec un taux de TP plus bas (p=0,015). Aucune différence significative n´a été retrouvée concernant le taux des leucocytes, des plaquettes et des phosphatases alcalines (Tableau 2).

**Tableau 2:** comparaison des caractéristiques biologiques entre les deux groupes

Paramètres biologiques	G1	G2	P
TP (%)	40	32,4	0,015
INR	2	3,6	0,015
Taux de la bilirubine (μmol/l)	157,45	332,67	0,0001
Taux des PAL (UI/L)	168,11	171,4	0,957
ALAT (UI/L)	1703	784	0,012
ASAT (UI/L)	1547	813	0,023
Taux de la créatinine (μmol/l)	60,5	102,9	0,003
Taux de l´urée (mmol/l)	5,4	10,4	0,007
Taux des leucocytes (/mm3)	8384,55	10166,67	0,343
Taux des plaquettes (/mm3)	330050	157026,67	0,234

TP: Taux de prothrombine, INR: International normalized ratio, PAL: Phosphatases alcalines, ALAT: Alanine aminotransférase, ASAT: Aspartate aminotransférase

Pour une valeur seuil de 2,45, l´aire sous courbe ROC du taux d´INR était de 0,711 [IC95%, 0,566-0,855] avec une sensibilité à 73,3% et une spécificité de 63,6%, avec p =0,017.

Pour une valeur seuil de 230,5 μmol/l, l´aire sous courbe ROC du taux de la bilirubine était de 0,780 [IC95%, 0,614-0,946] avec une sensibilité à 73,3% et une spécificité de 81,8%, avec p =0,0001.

Pour une valeur seuil de 60,5 μmol/l, l´aire sous courbe ROC du taux de la créatinine était de 0,612 [IC95%, 0,402-0,822] avec une sensibilité à 53,3% et une spécificité de 72%, avec p =0,049.

Pour une valeur seuil de 5,5 mmol/l, l´aire sous courbe ROC du taux d´urée était de 0,770 [IC95%, 0,604-0,935] avec une sensibilité à 80% et une spécificité de 72,7%, avec p =0,0001.

Pour le taux de la prothrombine, l´aire sous courbe ROC n´était pas significatif (l´aire sous courbe ROC = 0,310).

**Facteurs étiologiques:** la comparaison des étiologies entre les deux groupes a montré que seules l´hépatite auto-immune et la cause indéterminée étaient associées à l´apparition de l´EH avec une différence significative respectivement p=0.003 et 0,044. Il n´y avait pas de différence significative pour les autres étiologies (Tableau 3).

**Tableau 3:** comparaison des groupes selon les étiologies

Etiologies	G1	G2	P
HVA	13	2	0,137
HVB	16	2	0,068
HVC	4	0	0,298
HSV	1	0	0,741
HAI	0	4	**0,003**
SBC	1	1	0,386
Médicamenteuse	6	2	0,334
Cardiaque	2	0	0,553
Indéterminée	1	3	**0,044**
Tumorale	0	1	0,254

VHA: Virus de l´hépatite A, VHB: Virus de l´hépatite B, VHC: Virus de l´hépatite C, HSV: Herpès simplex virus, HAI: Hépatite auto-immune, SBC: Syndrome de Budd-Chiarri.

**Figure 2:**
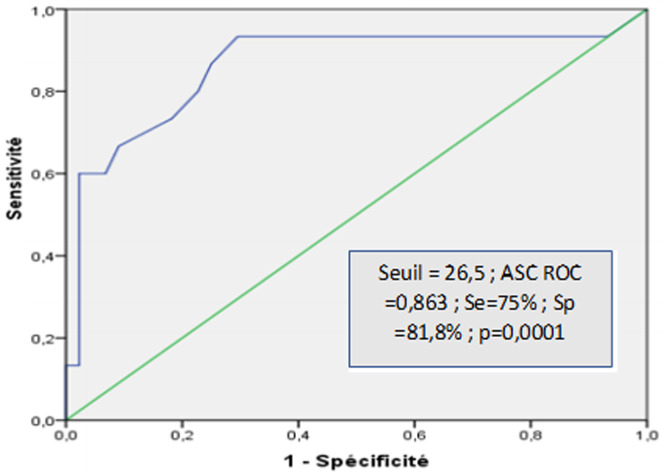
courbe ROC du score MELD pour l´évaluation du risque de l´encéphalopathie hépatique

**Comparaison selon le score MELD:** les patients ayant évolué à l´EH avaient un score MELD significativement plus élevé par rapport à celui du G1 (p=0,0001). La représentation graphique de la corrélation entre le score MELD et le risque de développement d´une EH a suggéré un risque plus important à partir d´une valeur de 26,5 (aire sous la courbe ROC = 0,863 [IC95%, 0,733-0,993] avec une sensibilité à 75% et une spécificité à 81,8%, p=0,0001) (Figure 2).

**Résultats de l´analyse multivariée:** en analyse multivariée, seule une étiologie auto-immune et un taux d´urée supérieur à 5,5 mmol/l étaient significativement associés à la survenue d´une EH, respectivement p=0,021 et 0,003. L´analyse multivariée est résumée dans le Tableau 4.

**Tableau 4:** résultat de l´analyse multivariée

Variables	P en univarié	P en multivariée
Délai de consultation supérieur à 9 jours	0,023	0,502
INR supérieur à 2,45	0,017	0,115
Taux de la bilirubine supérieur à 230,5 μmol/l	0,0001	0,136
Taux de MELD score supérieur à 26,5	0,0001	0,116
Taux d´urée supérieur à 5,5 mmol/l	0,0001	**0,03**
Taux de Créatinine supérieur à 60,5 μmol/l	0,049	0,550
Cause auto-immune	0,003	**0,021**
Cause indéterminée	0,04	0,646

INR: International normalized ratio, MELD: Model for End-stage Liver Disease

## Discussion

L´apparition d´une EH au cours de l´AHAS annonce la progression vers l´insuffisance hépatique aiguë et représente un tournant évolutif. En effet, l´apparition de l´IHA fait la gravité de la maladie en raison de l´association à une mortalité élevée, ce qui souligne l´importance d´une prise en charge précoce avant l´apparition de cette complication. Le pourcentage de progression vers une EH varie entre 19% et 31% [6,7]. Dans notre étude, 25% de nos patients ont évolué vers une EH.

La survenue d´une EH était plus fréquente chez les patients les plus âgés. En effet, Takikawa *et al*. ont établi que l´âge avancé est un facteur de risque important pour prédire l´EH [7]. Ceci a été conforté par plusieurs autres études [6,8,9]. Dans notre série, l´âge moyen des patients ayant développé une EH était plus élevé sans différence significative (p=0,143). Concernant le rôle du genre, notre résultat concorde avec la littérature. En effet, plusieurs études n´ont pas retenu le sexe comme étant un facteur de risque de l´EH [6,9].

L´alcoolisme chronique était associé à la survenue d´une EH selon Kim *et al*., Schiødt *et al*. (respectivement = 0,049 et 0,01) [10,11]. Contrairement à notre étude, l´alcoolisme était plus fréquemment retrouvé chez les patients n´ayant pas présenté une EH (p=0.039). Concernant le diabète, certaines études n´ont pas trouvé ce facteur comme prédictif de l´EH [8,10] , contrairement à l´étude de Hye Sun Shin *et al*. où le diabète était associé à l´apparition de l´IHA [9]. Dans notre étude, le diabète n´était pas un facteur prédictif de l´EH.

Concernant le délai d´apparition des symptômes, un délai prolongé de consultation était parmi les facteurs prédictifs de la survenue de l´EH selon Koch *et al*. [6]. Cependant, dans une autre étude chinoise de Xiong *et al*. [8], la durée moyenne d´évolution des symptômes était comparable entre les 2 groupes (p=0,112). Dans notre série, la comparaison du délai de consultation par rapport à l´apparition des symptômes entre les deux groupes a montré un délai plus long chez les patients qui ont présenté l´EH avec une différence significative p=0.004 en univarié. Cela pourrait être expliqué par l´effet d´une prise en charge plus rapide et l´instauration des mesures thérapeutiques générales et étiologiques.

La gravité de la coagulopathie et l´élévation de la bilirubinémie représentent également des facteurs prédictifs importants du risque d´évolution vers une EH [6-9,12]. Dans notre étude, la survenue de l´EH était statistiquement associée à un taux d´INR supérieur à 2,45 et un taux de bilirubine supérieur à 230,5 μmol/L à l´analyse univariée (respectivement p=0,017; p=0,0001). En ce qui concerne la fonction rénale, MacKinney *et al*., en étudiant les caractéristiques de l´hépatite aiguë A, ont considéré qu´une élévation de la créatinine au-dessus de 2 mg/dl comme étant un facteur prédictif de survenue d´une EH [13]. Cependant, dans l´étude américaine de Koch *et al*., le taux de la créatinémie n´était pas identifié comme étant un facteur prédictif d´EH (p=0,456) [6]. Dans notre étude, le taux moyen de la créatinine était significativement plus élevé chez les patients ayant évolué vers l´EH (p = 0,003). Le cut-off retrouvé était de 60,5 μmol/l avec une sensibilité de 53% et une spécificité de 72%. Le taux moyen de l´urée était significativement plus élevé chez les patients ayant présenté une EH avec une différence significative en analyse univariée (p=0,007) et multivariée (p=0,003). Le cut-off retrouvé était de 5,5 mmol/l avec une sensibilité de 80% et une spécificité de 72%. Le taux d´urée et son impact évolutif au cours de l´AHAS n´a pas été abordé dans la littérature.

L´origine étiologique joue un rôle déterminant dans le pronostic de l´AHAS et sa progression vers une IHA. En effet, les hépatites virales ont un faible potentiel évolutif à l´EH avec un risque aux alentours de 1 à 4% [8,10,14-16]. L'incrimination de l´infection virale C aiguë dans la survenue d´une EH est exceptionnelle et limitée à quelques rares cas, surtout en l´absence d´une hépatopathie sous-jacente associée [14,17-19]. L´IHA induite par le VHE est rare, sauf chez les femmes enceintes au troisième trimestre où elle peut atteindre jusqu´à 25% des patientes [14,15,20]. Contrairement aux causes virales, l´hépatite auto-immune dans sa présentation aiguë, les causes médicamenteuses non dues au paracétamol et la cause indéterminée ont un risque important évolutif vers l´IHA, ce qui incite une vigilance particulière face à ces étiologies [6,7]. L´hépatite auto-immune dans sa forme aiguë représente un haut potentiel d´évolution vers l´IHA en touchant prés le tiers des malades [7,21]. Dans notre série, l´étiologie auto-immune de l´AHAS était un facteur prédictif de survenue d´une EH en analyse univariée et multivariée. L´étiologie indéterminée de l´insuffisance hépatique aiguë atteint dans certaines séries 30 à 40% des cas [22]. Dans notre série, une étiologie indéterminée était un facteur prédictif de survenue d´une EH en analyse univariée (p=0,044).

Le score MELD est un score utilisé classiquement dans les cirrhoses pour sélectionner les malades en attente de transplantation hépatique et pour évaluer la sévérité de la maladie [23]. Une étude coréenne colligeant 304 patients ayant pour but l´évaluation du risque d´IHA et de la mortalité au cours de l´hépatite aiguë A. Le score MELD était associé à la survenue de l´EH en analyse uni et multivariée. Ils ont retrouvé qu´un score MELD supérieur ou égal à 23,5 semblait être associé au développement de l´IHA chez les patients atteints d´hépatite aiguë A [9]. Lars E *et al*. ont étudié l´apport du score MELD pour prédire la survenue d´EH et la mortalité chez 460 patients présentant une intoxication au paracétamol. Ils ont conclu que le score MELD était significativement plus élevé chez les patients qui ont développé une EH [24]. Dans notre étude, le score MELD était un facteur associé à la survenue d´une EH en analyse univariée (p < 0,0001). Un score MELD supérieur à 26,5 était le cut-off retrouvé avec une sensibilité de 75% et spécificité de 81%. De ce fait, le score MELD pourrait être utile comme score prédictif d´EH chez les patients admis avec une atteinte hépatique sévère. Des séries plus larges demeurent indispensables pour valider l´utilité de ce score. L´équipe de l´hôpital de Beaujon en France a proposé une stratégie de prise en charge précoce avec un transfert à un centre spécialisé avant l´apparition d´EH selon les facteurs de risque retrouvés dans la littérature [25]. Ces critères sont résumés dans le Tableau 5.

**Tableau 5:** critère de transfert à un centre de transplantation hépatique avant l´apparition de l´encéphalopathie hépatique selon l´équipe de Beaujon

Taux de TP (%)	Caractéristiques des malades
**50% et 30%**	Au moins un critère requis: -Age <15 ans -Age > 40 ans avec étiologie défavorable* -Fièvre > 38 ou étiologie rare** (en dehors des atteintes au paracétamol) -Contexte post opératoire -Grossesse -Hépatopathie chronique sous-jacente -Comorbidités sous-jacentes à risque: diabète, VIH, Malaria, cancer, insuffisance rénale aiguë sévère -Hyperbilirubinémie > 250 μmol/L
**<30%**	Atteinte hépatique aiguë non au paracétamol surtout si: Âge> 40 ans ou étiologie défavorable**

* Les étiologies défavorables considérées: les causes médicamenteuses et indéterminées selon les critères de King´s college [1] et les causes indéterminée, auto-immune, et la réactivation virale B aiguë selon Takikawa [7]. **Les étiologies inhabituelles: maladie de Wilson, syndrome de Budd-chiarri et le syndrome de Reye´s post aspirine.

**Les limites:** la première limite est le fait que cette étude est rétrospective, cela implique l'existence de quelques données manquantes, la deuxième limite est le caractère monocentrique réduisant considérablement le nombre de patients et par conséquent la puissance des résultats. Cependant, ces limites n’enlèvent rien à la qualité des renseignements fournis par cette étude.

## Conclusion

L´AHAS est une pathologie certes rare mais grave. Le principal facteur pronostique est la progression vers une IHA ce qui souligne l´importance de déterminer les facteurs prédictifs de l´EH au cours de l´AHAS afin d´identifier précocement les patients à risque d´une évolution défavorable à partir de simples tests de routine comme la sévérité de la coagulopathie, le taux de la bilirubine et la fonction rénale. L´étiologie en cause joue aussi un rôle important. Les causes virales, les plus fréquentes dans notre pays, ont un faible potentiel évolutif à l´EH, contrairement à l´hépatite auto-immune dans sa présentation aiguë et la cause indéterminée. Cependant, d´autres études plus larges et multicentriques sont nécessaires pour établir un score faisant intervenir l´ensemble des facteurs de risque pour prédire avec précision le risque d´EH.

### Etat des connaissances sur le sujet

- Le principal facteur pronostique au cours des atteintes hépatiques aiguës sévères est l´apparition de l´encéphalopathie hépatique;
- L´insuffisance hépatique aiguë est un état de défaillance hépatique aiguë compliqué d´une EH, associé à coagulopathie sévère et une détérioration systémique résultant d´un syndrome de réponse inflammatoire systémique avec un taux de mortalité élevé;
- L´apparition d´une encéphalopathie hépatique dépend de l´association de plusieurs facteurs essentiellement l´intensité des troubles de la coagulation, le taux de la bilirubine et l´étiologie.

### Contribution de notre étude à la connaissance

- Dans notre pays, l´étiologie de l´atteinte hépatique aiguë sévère est dominée par les causes virales; la fonction rénale, particulièrement le taux de l´urée, est un facteur associé à l´apparition d´une encéphalopathie hépatique au cours de l´atteinte hépatique aiguë sévère;
- La détermination des facteurs prédictifs de l´encéphalopathie hépatique au cours de l´atteinte hépatique aiguë sévère à partir de simples tests de routine permet à aider le clinicien à sélectionner les patients à risque d´une évolution défavorable;
- Le score MELD pourrait être contributif pour prédire l´encéphalopathie hépatique chez les patients admis avec une atteinte hépatique aiguë sévère; des séries plus larges demeurent indispensables pour valider l´utilité de ce score.
